# Mechanical, Microstructural and Drying Shrinkage Properties of NaOH-Pretreated Crumb Rubber Concrete: RSM-Based Modeling and Optimization

**DOI:** 10.3390/ma15072588

**Published:** 2022-04-01

**Authors:** Pretta Malaysia Appana, Bashar S. Mohammed, Isyaka Abdulkadir, M. O. A. Ali, M. S. Liew

**Affiliations:** 1 Civil and Environmental Engineering Department, Faculty of Engineering, Universiti Teknologi PETRONAS (UTP), Seri Iskandar 32610, Perak, Malaysia; pretta_25146@utp.edu.my (P.M.A.); isyaka_18000638@utp.edu.my (I.A.); montasir.ahmedali@utp.edu.my (M.O.A.A.); shahir_liew@utp.edu.my (M.S.L.); 2 Civil Engineering Department, Bayero University, Kano 700241, Nigeria

**Keywords:** crumb rubber (CR), pretreatment, response surface methodology (RSM), CR-concrete, optimization

## Abstract

One of the primary causes of the low mechanical properties of rubberized concrete is the weak bond between crumb rubber (CR) and hardened cement paste. Many CR pretreatment techniques have been researched in an attempt to mitigate this problem. The NaOH pretreatment method is one of the most widely used, although the reported results are inconsistent due to the absence of standardized NaOH pretreatment concentrations and CR replacement levels. This study aims to develop models for predicting the mechanical and shrinkage properties of NaOH-pretreated CR concrete (NaOH-CRC) and conduct multi-objective optimization using response surface methodology (RSM). The RSM generated experimental runs using three levels (0, 5, and 10%) of both NaOH pretreatment concentration and the CR replacement level of fine aggregate by volume as the input factors. At 28 days, the concrete’s compressive, flexural, and tensile strengths (CS, FS, and TS), as well as its drying shrinkage (S), were evaluated as the responses. The results revealed that higher CR replacements led to lower mechanical strengths and higher shrinkage. However, the strength loss and the shrinkage significantly reduced by 22%, 44%, 43%, and 60% for CS, FS, TS, and S, respectively, after the pretreatment. Using field-emission scanning electron microscopy (FESEM), the microstructural investigation indicated a significantly reduced interfacial transition zone (ITZ) with increasing NaOH pretreatment. The developed RSM models were evaluated using ANOVA and found to have high R^2^ values ranging from 78.7% to 98%. The optimization produced NaOH and CR levels of 10% and 2%, respectively, with high desirability of 71.4%.

## 1. Introduction

The ever-increasing world population raises transportation demands, which lead to increased automobile production. This has also resulted in an unprecedented increase in global waste tire generation [1,2,3]. It is estimated that one vehicle tire is discarded per capita in developed nations, culminating in 1 billion discarded waste tires each year [4,5]. The volume is expected to escalate due to increased traffic, with annual disposed waste tires reaching 5 billion by 2030. The negative consequences of indiscriminate waste tire disposal include, among other things, the loss of usable land, a rise in pests and rodents, and the likelihood of an uncontrollable fire outbreak with dangerous smoke emissions [6,7,8,9]. Furthermore, the tires emit harmful leachate into the earth, polluting the land and water around them [10].

Crumb rubber (CR) is produced from waste tires using a milling process that results in rubber particles ranging in size from 0.425 to 4.75 mm. The nature of the particles is determined by the temperature and milling method employed (cracker mill, granular mill, or micro mill process) [5]. Its usage in concrete has been researched for more than three decades, as evident from reported work on its use as aggregate by Eldin and Senouchi [11]. Many research findings revealed that the use of tire rubber in cement composites has many advantages such as enhanced impact and energy absorption capacity [12,13,14,15,16], improved tensile and ductility behavior [7,17,18], increased abrasion resistance [19], better freeze–thaw resistance [16,20,21,22,23], enhanced sound and insulation behavior among others [24]. As a consequence of these factors, there has been an increase in research on cementitious composites incorporating CR to solve the environmental challenge and produce concrete with remarkable properties.

Despite the observed improvements in properties of concrete due to the incorporation of CR, it is universally acknowledged that mechanical strengths are adversely affected. Among the reasons given for the decline in strength with an increase in CR is its lack of proper bonding with the hardened cement matrix [25,26,27,28]. The poor bonding is caused by the rubber’s hydrophobic property, which repels water during mixing, trapping air on its surface [29,30]. Other reasons include the softer nature of the CR particles compared with the replaced aggregates and the increased porosity of the concrete owing to the high air content [29]. Many researchers have attempted to improve the bonding by subjecting the CR to various pretreatments. The most recent of these efforts is by Abdulkadir et al. [29] who investigated a novel use of different concentrations of graphene oxide (GOC) for CR pretreatment used in engineered cementitious composite (ECC). Their findings revealed that GO enhanced the bonding between the CR and cement paste because of its high reactivity due to many reactive functional groups on its surface. At 5% GO-CR replacement, there was a 50.3%, 70.4%, and 68.3% improvement in the composite’s compressive, flexural, and tensile strengths compared to the control mix with 0 mg/mL GOC and the mix with 1.0 mg/mL GOC, respectively. However, GO is significantly more expensive than NaOH, and this is the first published study on its usage as a CR pretreatment agent; thus, additional research is needed to confirm its benefits. Other drastic measures taken include a unique method of thermal pretreatment of CR developed by Abd-Elaal et al. [31]. The authors reported a 60.3% strength recovery at 20% thermally treated CR replacement. Also, Gregorio and Adam [32] used an ultraviolet (UV) method of CR pretreatment to enhance the bonding between the CR and the hardened cement paste. Samples containing UV-CR had only a 6% lower strength than control concrete containing no CR.

Despite the reported improvements by the methods mentioned above, the most popular technique of modifying the CR surface for better bonding with the cement matrix is an alkaline pretreatment using NaOH. Many researchers have used this pretreatment method with varying degrees of enhancement of the fresh and mechanical properties of the composites. Ameri et al. [25] reported a 59% increase in strength of rubberized mortar after pretreatment with NaOH. However, they reported a decline in the workability of the mortar after 24 h of the CR-alkali pretreatment. Similarly, Youssf et al. [27] showed that the NaOH pretreatment performed better than other chemicals (H_2_O_2_, CaCl_2_, H_2_SO_4_, Silane, and KMnO_4__NAHSO_4_). However, they concluded that the use of water washing as a form of CR pretreatment performed much better than all the chemical pretreatment methods, and the improvement using the chemical pretreatment is not worth the cost. Furthermore, Safan et al. [33] reported improved compressive, flexural, and tensile strength of concrete containing CR treated with 15%, 20%, and 30% NaOH. They concluded that 20% pretreatment gave a better performance. Also, the NaOH pretreatment improves the durability of the rubberized concrete by exhibiting better freeze and thaw resistance and higher resistance to expansion due to alkali-silica reaction (ASR) [34]. In the same vein, similar works utilizing NaOH pretreatment solely or in combination with other methods were reported in the following articles: [35,36,37,38].

The NaOH works on the CR physically and chemically. The physical process includes cleaning the CR surface of all debris and grime and etching its surface to improve the physical bonding with the hardened cement paste [35]. The chemical procedure involves the conversion of the rubber’s hydrophobic zinc stearate into a hydrophilic and water-soluble sodium stearate that can be readily washed off.

There are several variabilities in the reported improvements in the mechanical properties of cementitious composites using NaOH pretreated CR. This can be attributed to the varying NaOH concentrations and treated CR replacements employed in numerous studies. Hence, there is a need to conduct more research to establish the exact influence of NaOH pretreatment on crumb rubber concrete (CRC) properties and obtain an optimized concentration and CR replacement level to produce a CRC with desirable properties. The study aims to develop empirical models for predicting the properties of NaOH pretreated CR concrete (NaOH-CRC). In addition, a multi-objective optimization will be carried out to obtain the optimal amounts of NaOH and CR that can give the most preferred properties of the concrete using response surface methodology (RSM).

## 2. Materials and Methods

### 2.1. Materials

**Figure 1:**
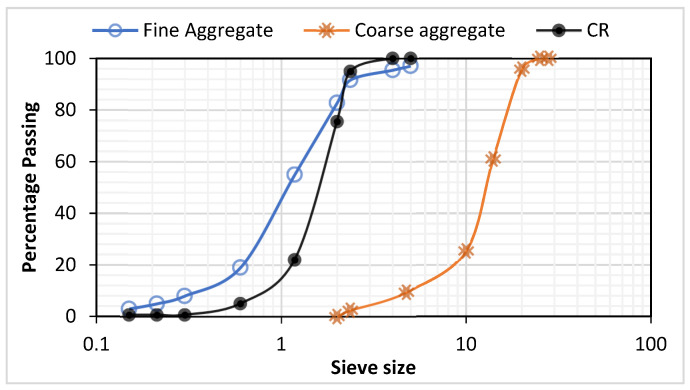
Grading curves for the aggregates and crumb rubber (CR).

The ingredients used include type I ordinary Portland cement conforming to the specifications of ASTM C150 and having the properties and oxide composition presented in Table 1. The fine and coarse aggregates used conform to the specifications of ASTM C33 (ASTM, 2055i) and have a specific gravity of 2.65 and 2.61, respectively. The CR used to replace the fine aggregate has a specific gravity of 0.95 and a fineness modulus of 0.92. The CR particle size ranged from 1 mm to 5 mm, as shown in Figure 1. Figure 1 presents the combined graph for the grading curves of the aggregates and CR used. NaOH of 95 to 98% chemical purity grade was used for the pretreatment. Clean tap water conforming to the specifications of ASTM C1602/C1602 M was used for mixing and curing. In order to achieve a workable concrete at a reasonably low water-cement ratio of 0.47, a Sika Viscocrete-2044^®^ superplasticizer (SP) was used. The SP has a 0.1% free chloride content, a specific gravity of 1.08, a PH of 6.2, and a dose of 1% of cement weight was utilized for all mixtures.

### 2.2. Crumb Rubber (CR)-NaOH Pretreatment

**Figure 2:**
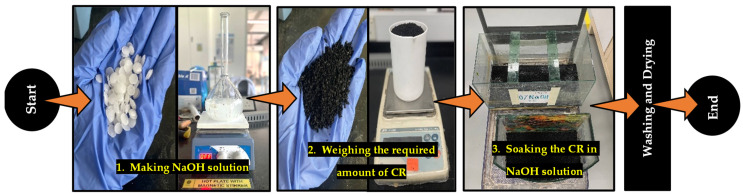
CR pretreatment process.

The pretreatment process is presented in Figure 2. This procedure was adopted from previous works on the use of NaOH for CR pretreatment [27,35,38]. In this research, three levels of NaOH pretreatment concentrations were considered. The first level is the control group, where the CR was used without any form of pretreatment considered as 0% concentration. The second and third groups were those pretreated with 5% and 10% solutions of NaOH, respectively. The process started by making a solution of the desired concentration. The CR was then soaked in the solution for 24 h, followed by washing with tap water until the PH became neutral. This step was followed by air drying the washed CR at an ambient temperature. The dried pretreated CR was then used in the concrete mix based on the generated RSM mix proportions.

### 2.3. Response Surface Methodology (RSM) Design and Mix Proportion

RSM is a statistical approach in which input factors (independent variables) are assessed through output factors known as the responses (dependent variables). The analysis entails designing a series of experiments and collecting the empirical data as responses, followed by building response surface numerical models to validate the accuracy and optimizing the variables to fulfill the target responses [6,9]. In this research, the independent variables considered were three levels (0, 5, and 10%) of the NaOH pretreatment percent concentration and fine aggregate CR replacement levels by volume. The responses considered were the compressive (CS), flexural (FS), tensile (TS) strengths, and shrinkage (S). Applying the central composite design of the RSM, and with two repetitions of the central points, nine mixes with varied combinations of the input variables were produced, as shown in Table 2.

The proportions of other materials were used as adopted from previous research. The cement, coarse aggregate, and water contents were kept constant for all mixes, as shown in Table 2. The water:cement ratio used was 0.47.

### 2.4. Concrete Mixing and Samples Preparation

The concrete was mixed with a pan-type mixer having a double rotation mechanism. The mixing sequence followed is similar to that used by Youssf et al. [27]. The process began with mixing the fine and coarse aggregates with the CR and half of the mixing water for 1 min. The mix was allowed to rest for 2 min, followed by adding the cement and the remaining water with a plasticizer and mixing this for 2 to 5 min.

**Figure 3:**
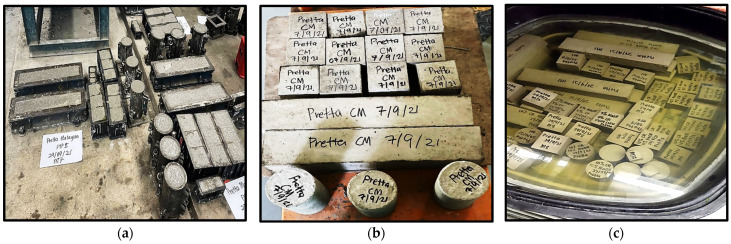
(**a**) Concrete samples after casting. (**b**) Demoulded samples ready for curing after labeling. (**c**) Test samples in curing tank.

The fresh NaOH-CRC was then cast into lightly oiled moulds for various tests, as shown in Figure 3a. The samples were removed from the moulds after 24 h, labeled accordingly as shown in Figure 3b, and put in the curing tank containing water at ambient temperature and pressure (Figure 3c).

### 2.5. Sample Testing

#### 2.5.1. Compressive Strength

For each mix, three 100 mm cube samples were tested for compressive strength at 28 days, and the average of the results was reported. The CS test was performed based on the provisions of BS EN 12390-3.

#### 2.5.2. Flexural Properties Test

**Figure 4:**
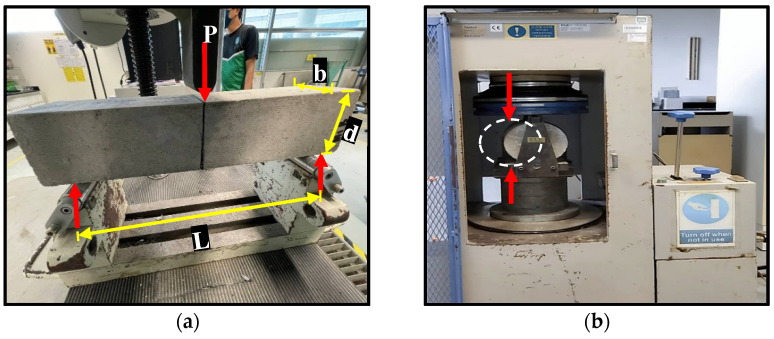
(**a**) Three-point loading flexural test. (**b**) Splitting tensile strength test.

The flexural test was conducted on two samples for each mix using the three-point loading test prescribed by BS EN 12390-5:2019. The test setup is shown in Figure 4a. Using a universal testing machine (UTM) of 200 kN capacity, a center point load was gradually applied on the sample at a 5 mm/min rate until failure. The load and mid-span deflection was recorded via an inbuilt data logger which feeds the test data to a computer that displays the readings in real time.

#### 2.5.3. Tensile Properties Test

For the tensile strength, cylinder samples having dimensions of 200 mm height and 100 mm diameter were subjected to a splitting tensile strength test based on the requirements of BS EN 12390-6:2019. The cylinder samples were laid horizontally within the testing jack, as shown in Figure 4b, and a continuous load was applied without shock at a rate of about 14 to 21 kg/cm^2^/min until failure. For each mix, the test was conducted on three cylinder specimens and the average was reported as the splitting tensile strength of the mix.

#### 2.5.4. Shrinkage Measurement

Cylinder samples were cast and marked for the determination of the drying shrinkage. The shrinkage measurements were taken every day for the first week, then weekly for the remaining three weeks. The shrinkage of each mix was calculated at the end of the measuring period and reported accordingly.

#### 2.5.5. Field-Emission Scanning Electron Microscopy (FESEM)

FESEM analysis was performed on samples from the mixes to gain an insight into the influence of the pretreatment at micro and nanoscale levels and better understand the interaction between the CR and the hardened matrix. The analysis is to obtain information on the interfacial transition zone (ITZ) between the CR and the hardened cement matrix. The imaging was performed using Supra 55VP microscope with ultrahigh-resolution capacity. Test samples of about 10 mm were cut from the specimens from each mix and coated with a gold sputter to enhance the images’ resolution.

## 3. Results and Discussion

### 3.1. Compressive Strength (CS)

**Figure 5:**
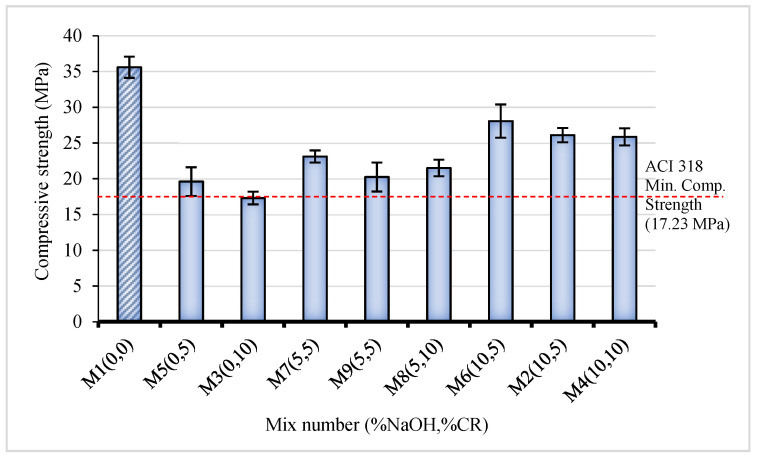
Compressive strength of NaOH-CRC mixes at 28 days.

Figure 5 presents the 28-day CS of the NaOH-CRC. There is an overall reduction in strength with an increase in CR replacement. The CS ranges between 17.29 and 35.6 MPa. Hence, all the mixes can be used for structural applications based on the ACI 318 specification on structural concrete, as indicated by the red line in Figure 5. The control mix (M1), having 0% NaOH and 0% CR as expected, exhibited the highest CS value of 36.5 MPa. With the replacement of 5% and 10% untreated CR (M5 and M3), the CS dropped by 45% and 51% compared to the control, respectively. However, when 5% NaOH (M7) pretreated CR was used for the replacement, the strength loss recorded was 35%, 10% lower than the strength loss recorded for 5% untreated CR (M5), representing a 22% lower reduction. Similarly, using 10% replacement of 5% NaOH pretreated CR shows an increase in strength of 11% compared to using untreated CR at the same replacement level. When the pretreatment concentration increased to 10% NaOH, the rate of strength loss significantly reduced, as can be observed from M6 (10% NaOH and 5% CR) exhibiting the highest CS following the control (M1) with just a 21% loss. In the same vein, despite having a higher CR content, M4 (10% NaOH, 10% CR) has a higher CS value than M5 (0% NaOH, 5% CR), M7, and M9 (5% NaOH, 5% CR). Increases of 43% and 51% were recorded between mixes containing 5% and 10% untreated CR and those containing 5% and 10% CR pretreated with 10 NaOH, which is greater than the improvements reported (8–40%) in most literature utilizing the same amount of NaOH pretreated CR [28]. This is due to the higher NaOH concentration than in the other mixes. As per the trend, the NaOH pretreatment significantly improves the CS. Despite having the same CR content, mixes with more NaOH concentrations have higher CS.

The NaOH pretreatment of CR enhances the CS of the concrete by enhancing the bonding between the CR and the cement paste. This is achieved through the physical and chemical interaction of the alkali with the rubber surface. The physical action of NaOH on the CR surface involves washing away all dirt and grease and etching the surface, making it cleaner and rougher, respectively, resulting in better bonding at the interface. On the chemical effect, the NaOH reacts with the zinc stearate present on the surface of the CR, converting it to sodium stearate. Zinc stearate is responsible for the hydrophobic nature of the CR, while sodium stearate is water-soluble and is washed up during the pretreatment process, thereby enhancing better bonding at the interfacial transition zone (ITZ) between the CR and the hardened cement matrix. The favorable effect of the NaOH pretreatment on the CS observed in this research is consistent with the findings of other studies [14,15,16].

**Figure 6:**
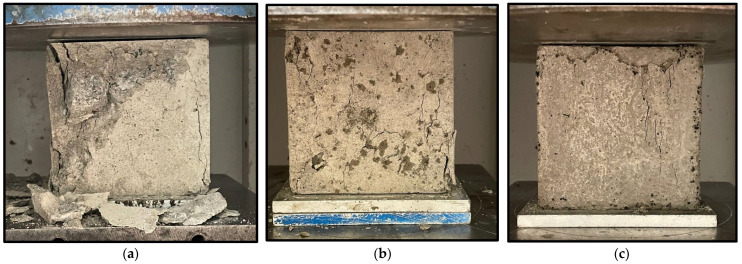
Failure mode of NaOH-CRC. (**a**) Control (M1-0% NaOH, 0% CR). (**b**) M3 (0% NaOH, 10% CR). (**c**) M6 (10% NaOH, 5% CR).

The presence of the CR influences the failure pattern of the samples. As the CR increased, the brittleness of the mixes decreased. The control mix (M1) having 0% CR failed by developing many cracks and breaking into different fragments, as shown in Figure 6a. However, M3 containing 10% untreated CR failed in a more ductile mode than M1, as shown in Figure 6b. As the NaOH pretreatment increased, the crack patterns also reduced significantly. This can be observed from Figure 6c, showing the failure mode of M6 having 5% NaOH and 10% CR. This is due to the enhanced bonding and better energy absorption due to NaOH and the effect of CR, respectively.

### 3.2. Flexural Behavior

**Figure 7:**
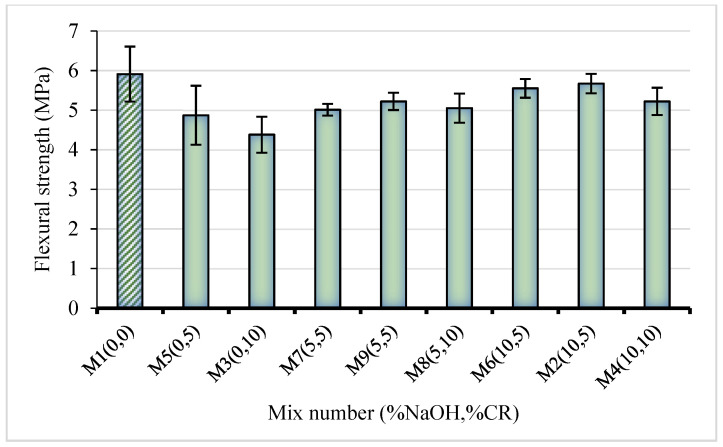
Flexural strength of NaOH-CRC mixes at 28 days.

The flexural strength (FS) result obtained from the three-point test conducted on the NaOH-CRC is presented in Figure 7. Similar to the CS result, an increase in the CR replacement affects the FS negatively. This is in agreement with the findings of other researchers. However, the pretreatment reduces the strength loss with CR replacement. The mix having the highest FS (5.91 MPa) is the control (M1), having 0% NaOH and 0% CR. With 5% and 10% untreated CR replacements, the FS was reduced by 17.6% and 26%, respectively, compared with the control. However, when the 5% NaOH pretreated CR was used at 5% and 10% replacements, the strength loss reduced to 11.7% and 14.6%, respectively. As the NaOH pretreatment concentration increased to 10%, the strength loss reduced significantly to 4% and 11.7% for 5% and 10% CR replacement levels. This represents a 16.4% and 19.2% increase in the flexural strength between samples with untreated CR and those with 5% and 10% NaOH pretreated CR, respectively. This is in sharp contrast to Youssf et al. [27] who reported that there was no difference in the flexural strength between a concrete with untreated CR and that with NaOH pretreated CR. Also, the enhancement in the flexural strength is more than that obtained using other pretreatment methods such as coating with cement paste, which only enhanced the flexural strength by 2% to 7%, as reported by most literature [30]. The improved FS with increased NaOH pretreatment is attributed to better adhesion between the CR and the cement paste. The lack of proper bonding and the wide ITZ led to low-stress transfer noticed with untreated CR. However, with pretreatment, the CR surface became more hydrophilic, leading to better bonding with cement paste and other cement hydration products, thus reducing the thickness of the ITZ.

### 3.3. Splitting Tensile Strength (TS)

**Figure 8:**
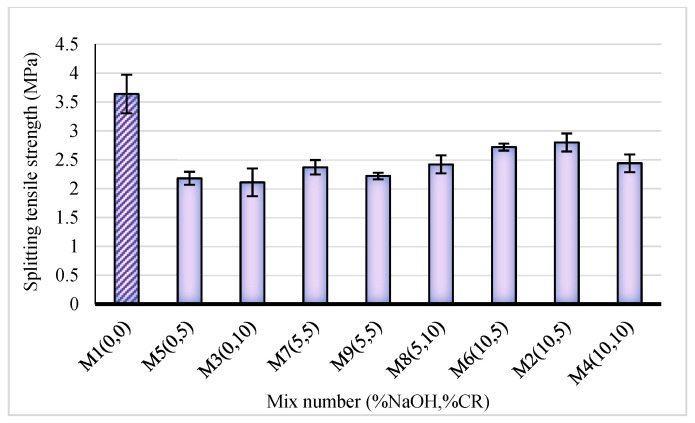
Splitting tensile strength of NaOH-CRC at 28 days.

Splitting tensile strength test entails exerting a diametric compressive load throughout a cylinder’s length until failure occurs. The tensile strength is then calculated from the failure load. The calculated tensile strength of the NaOH-CRC mixes is presented in Figure 8.

**Figure 9:**
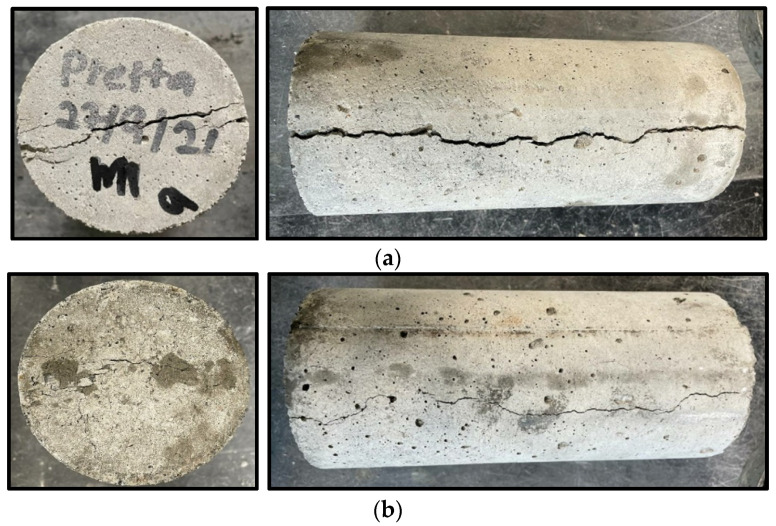
NaOH-CRC samples after splitting tensile test. (**a**) M1 (0% NaOH, 0% CR). (**b**) M2 (10% NaOH, 5% CR).

The TS followed the same pattern as the FS. There is a general decrease in the strength with the increase in the CR replacements. However, the strength loss was drastically reduced when using NaOH pretreated CR. The rate of decrease in the strength loss is directly proportional to the NaOH concentration. The strength values ranged from 2.11 MPa for the mix having the highest untreated CR content (M3) to 3.64 MPa for the control mix (M1). Compared to the control, the mix with the lowest strength loss is M2 (10% NaOH, 5% CR) with 23%. When compared to the control, the strength loss is 40% and 35% for the mixes with the same CR content but lower NaOH treatment [mix M7 (5% NaOH, 5% CR) and M5 (0% NaOH, 5% CR)]. Compared to the mixes having untreated CR (M5 and M3), there was a 24% and 32.7% increase in the tensile strength at 5% and 10% NaOH pretreatment (M6 and M2), respectively. This is in agreement with the reported enhancements of 3–36% in the splitting tensile strength reported from previous studies on NaOH pretreatment of CR [28]. Hence, it is safe to conclude that the pretreatment positively influenced the tensile performance of the NaOH-CRC. The pretreatment enhances the interaction of the CR within the composite. Similarly, the presence of CR had a significant impact on the mode of failure of the test samples. The control mix tends to have a wider crack and split than the mixes with CR, as shown in Figure 9a. This is due to the ability of the CR to absorb more energy, as explained by Murali et al. [39]. As the pretreatment concentration increased, the decrease in the ITZ due to the enhanced bonding between the CR and the cement paste increased the adhesion between the constituents making their separation harder, leading to minor crack development, as can be observed in Figure 9b.

### 3.4. Shrinkage

**Figure 10:**
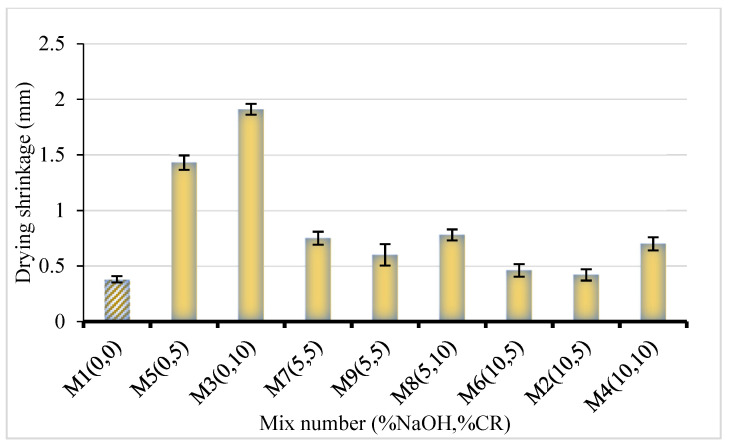
Shrinkage of NaOH-CRC.

The result of the drying shrinkage is shown in Figure 10. The shrinkage is directly proportional to the CR replacement level and inversely proportional to the NaOH pretreatment. As the concrete loses water, the cement paste shrinks in volume. The amount of restraint provided to the cement paste influences the change in the volume. However, the CR being softer than the surrounding materials can easily deform, allowing for the shrinkage to increase, as observed in this result with increasing CR. This finding is consistent with the findings of previous studies on the effect of CR on the shrinkage of cementitious composites [9]. However, with the NaOH pretreatment, more cement hydration products were produced at the interface, thereby densifying the concrete and making the ITZ more compact and less prone to shrinkage. This can be seen from the results as samples having higher NaOH pretreatment have lower shrinkage values and vice-versa.

### 3.5. Field-Emission Scanning Electron Microscopy (FESEM) Analysis

**Figure 11:**
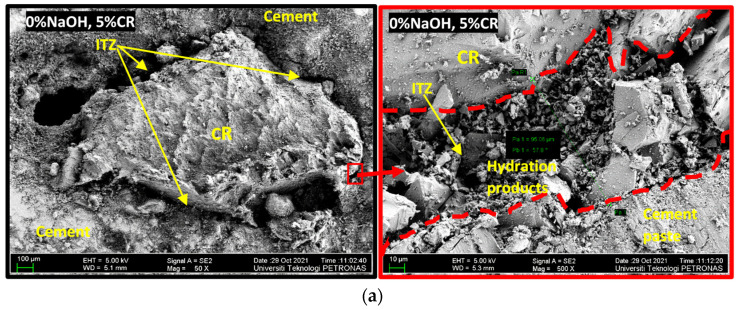

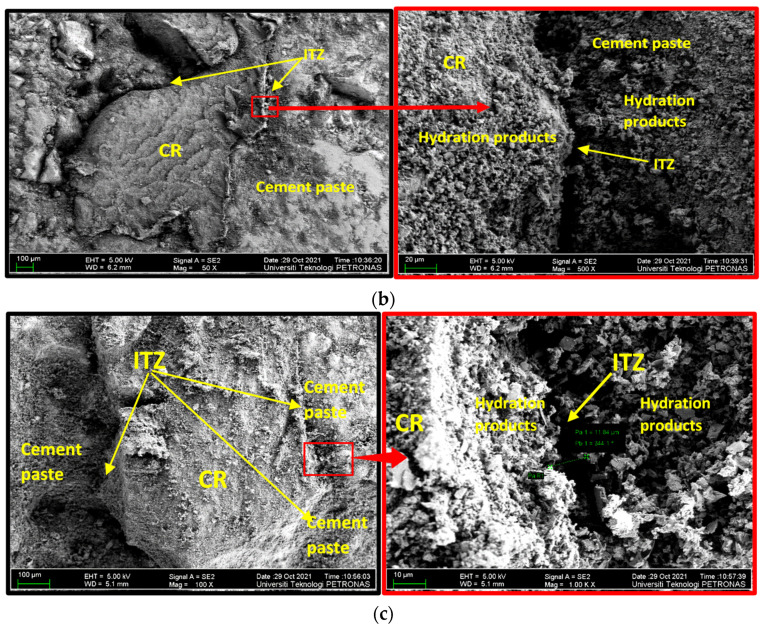
Field-emission scanning electron microscopy (FESEM) images for NaOH-CRC (**a**) M5 (0% NaOH, 5% CR) (**b**) M7 (5% NaOH, 5% CR) (**c**) M2 (10% NaOH, 5% CR).

A FESEM investigation was carried out to assess the influence of the NaOH pretreatment on the CR and how it affects the concrete’s properties. Figure 11 shows the FESEM images of some of the samples. Figure 11a shows a mix (M5) containing untreated CR at 5% replacement level. As can be observed, the ITZ between the CR and the hardened cement paste is wide due to the poor bonding between the two. An enlarged image of the ITZ is shown on the right as an inset in Figure 11a. A closer look at the ITZ shows the surface of the CR without the presence of cement hydration products. On the contrary, many of the hydration products can be observed at the edge of the hardened cement paste. This agrees with the result of the mechanical properties of the rubberized concrete, where samples containing untreated CR have the lowest strengths because of the wide ITZ and lower stress transfer.

On the other hand, Figure 11b shows a FESEM image of a mix (M7) containing a 5% CR pretreated with 5% NaOH. In sharp contrast to the sample presented in Figure 11a, there is a noticeable decrease in the size of the ITZ between the CR and hardened cement paste. A closer look at the magnified ITZ (inset) shows cement hydration products on both sides of the CR and the hardened cement paste. The presence of the hydration products on the CR surface confirms the effect of the NaOH pretreatment in enhancing the reactivity of the CR surface, which succeeded in decreasing the size of the ITZ. This impact is due to the change of the hydrophobic zinc stearate on the CR’s surface into a water-soluble sodium stearate that was washed away during the pretreatment process. This is similar to the effect of pretreatment of CR with sulphuric acid, where the zinc stearate was converted to zinc sulfate, as reported by Kashani et al. [34]. This effect became more apparent at higher NaOH pretreatment (M2), as displayed in Figure 11c. The ITZ became significantly smaller as compared to that of M5 and M7. As the ITZ reduces, the transfer of stress increases between the hardened cement paste and the CR, which translates to enhanced strength of the concrete. This is in line with the findings of Safan et al. [33] and Fernando et al. [38].

The rate of strength reduction increases with an increase in CR replacement levels for all the NaOH pretreatment concentrations. This is due to the soft nature of the CR particles, which act as artificial flaws within the concrete. However, the enhancing effect of the NaOH pretreatment can be observed from the mechanical strength results where all samples with higher NaOH concentration exhibited higher strengths than samples containing the same CR content but with lower NaOH pretreatment concentration.

## 4. RSM Analyses

### 4.1. Response Surface Models and Analysis of Variance (ANOVA)

RSM is used to develop models for response prediction as part of the study data analyses. The independent variables evaluated as input factors from the beginning are the NaOH pretreatment concentration and the CR replacement levels of fine aggregate by volume. The mechanical strengths (CS, FS, and TS) and the drying shrinkage (S) were the responses under consideration (output factors). Equations (1)–(4) present the developed response surface-based predictive models. These models are developed from the empirical data generated during the earlier experimental investigations. The models presented here are in coded factors. By default, high levels of the variables are encoded as +1, while low levels are encoded as −1. The coded equations may be used to identify the relative relevance of the factors by comparing their coefficients. As input factors, A and B represent the NaOH concentration and CR replacement levels. Quadratic models were found to be more suitable for the responses except for the FS that was fitted with a linear model.

(1) $$CS=+22.01+3.82\times A-8.53\times B+0.46\times AB+11.08\times A^{2}+7.35\times B^{2}$$

(2) FS=+5.33+0.31×A−0.56×B

(3) $$TS=+2.36+0.31\times A-0.87\times B-0.14\times AB+0.057\times A^{2}+0.80\times B^{2}$$

(4) $$S=+0.62-0.51\times A+0.64\times B-0.096\times AB+0.36\times A^{2}-0.37\times B^{2}$$

The next stage in the RSM analyses is model validation. The developed models were validated using ANOVA at a 95% confidence level (5% level of significance). Hence, any model or model term having a probability of less than 5% is statistically significant. The result of the ANOVA analysis is presented in Table 3. As can be observed, all the developed models are significant, with a probability value of less than 0.05. As for the model terms, A, B, and B^2^ are significant model terms in CS and TS models. A and B are significant model terms in the FS model. The significant model terms are A, B, and A^2^ in the S model. Furthermore, one of the indicators for an accurate model is an insignificant lack of fit *p*-value. The lack of fit *p*-value is used to assess the fitness of the developed models, and a value of more than 5% is desirable. Hence, in this case, all the developed models have a lack of fit *p*-value of more than 0.05, indicating the good fit of the models.

Similarly, the quality of the developed models was assessed using the R^2^ (coefficient of determination) value as one of the model validation parameters shown in Table 4. The high R^2^ values of 0.9709, 0.7872, 0.9677, and 0.9803 for the CS, FS, TS, and S indicate a high degree of correlation between predicted and experimental results and the adequacy of the models in predicting the responses with high accuracy [40]. Furthermore, the adequate precision value (Adeq. Presc.) measures a developed model’s signal-to-noise ratio, and a value of more than 4 is required for a robust model. In this case, all the developed models have an Adeq. Presc. of more than 4, indicating that the models are robust and can be used to navigate the design space effectively.

**Figure 12:**
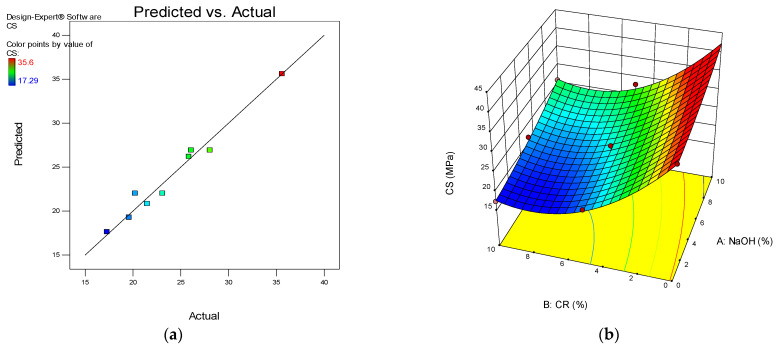
(**a**) Predicted vs. actual plot for compressive strength (CS). (**b**) 3D response surface diagram for CS.

**Figure 13:**
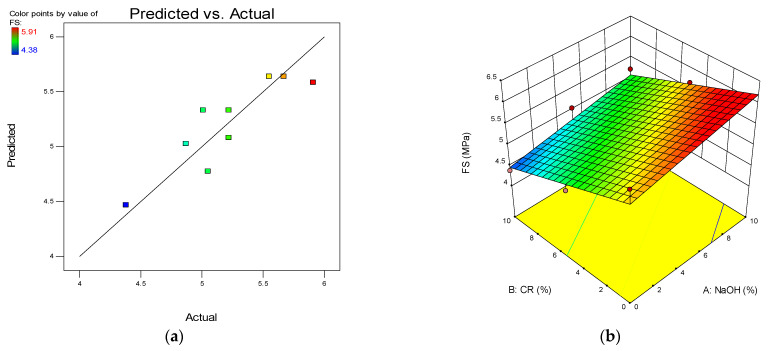
(**a**) Predicted vs. actual plot for flexural strength (FS). (**b**) 3D response surface diagram for FS.

**Figure 14:**
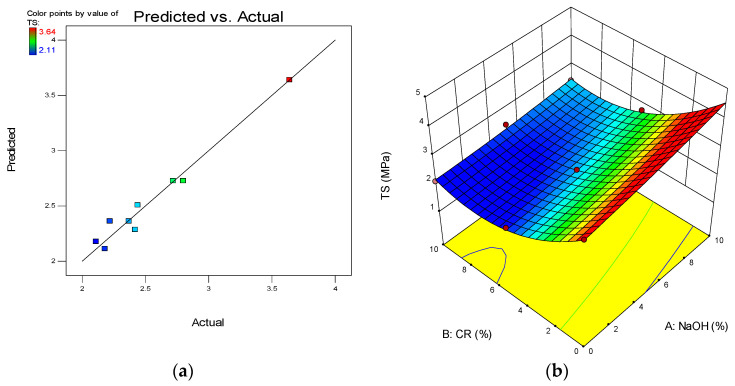
(**a**) Predicted vs. actual plot for tensile strength (TS). (**b**) 3D response surface diagram for TS.

**Figure 15:**
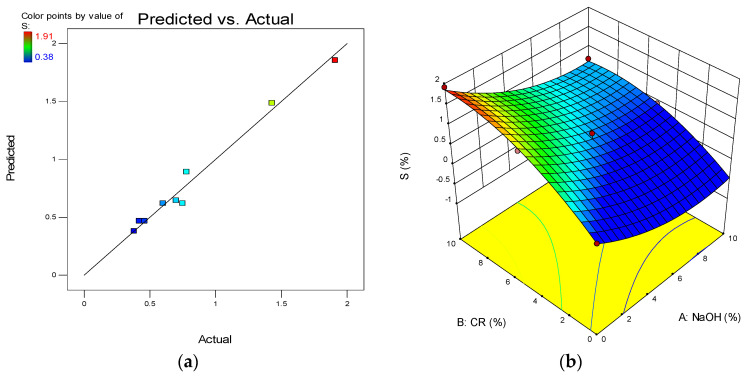
(**a**) Predicted vs. actual plot for S. (**b**) 3D response surface diagram for S.

One of the model diagnostic tools used to assess the quality of the developed models is the predicted vs. actual graph. The graphs depict the distribution of data points along the line of fit, and the linearity of the points around the lines of fit defines the model’s quality. The plot for all the developed models is presented in (a) parts of Figure 12, Figure 13, Figure 14 and Figure 15.

Another advantage of RSM is the development of the 3D response surface diagrams as shown in the (b) parts of Figure 12, Figure 13, Figure 14 and Figure 15 for CS, FS, TS, and S, respectively. These are model graphs that show the effect of the interaction of the input factors on the output factors. The graphs are expressed in contours that show the response variations at different levels and combinations of the independent variables. The magnitude of the responses is presented with a color gradient, usually the red regions indicating the highest values while the blue regions are indicating the lowest values. The patterns of the response surface plots in this research agree with the discussion of results given earlier in Section 3.

### 4.2. Multi-Objective Optimization

In order to determine the best amount of the NaOH concentration and the CR content that can give the most desirable properties of the NaOH-CRC properties under consideration (CS, FS, TS, and S), optimization was performed as part of the RSM analyses. Multi-objective optimization is more suitable as most real-life situations involve finding optimal solutions simultaneously for several objective functions. The goal, in this case, is to determine the optimal levels of the input factors that will yield optimal values of the responses simultaneously without compromising any.

The procedure begins with assigning the goal and importance to the input and output variables, followed by running the system and receiving the solutions as the optimal values of all the variables and the desirability index for the optimization. The desirability index gives the level of accuracy of the optimization process on a scale of 0 to 1 (0 to 100%). In this case, the optimization criteria are presented in Table 5. The goal is for the system to select between the lower and higher limits of the NaOH concentration (in range) while making the best use of (maximizing) the CR. As for the responses, the objective is to maximize all the mechanical strengths (CS, FS, and TS) while the drying shrinkage (S) should be minimized. Following the establishment of the objectives, the degree of importance for all variables was maintained at the default level of 3 (from a range of 1 to 5).

**Figure 16:**
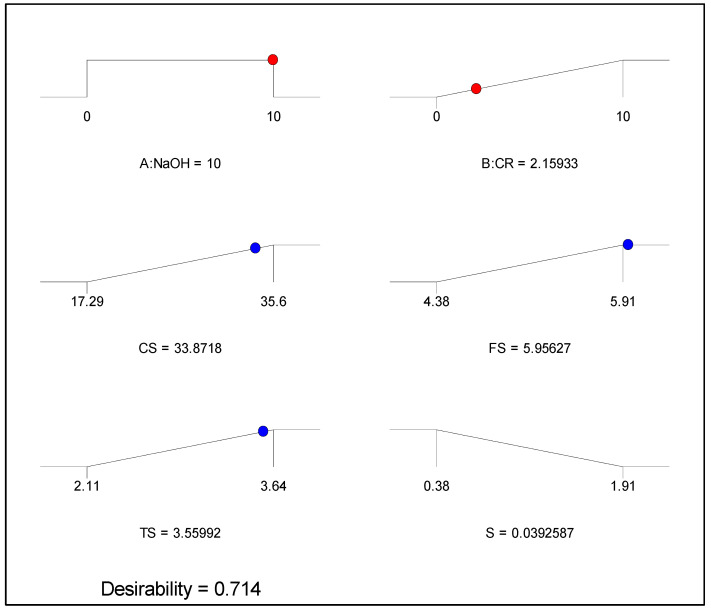
Optimization solution ramps.

**Figure 17:**
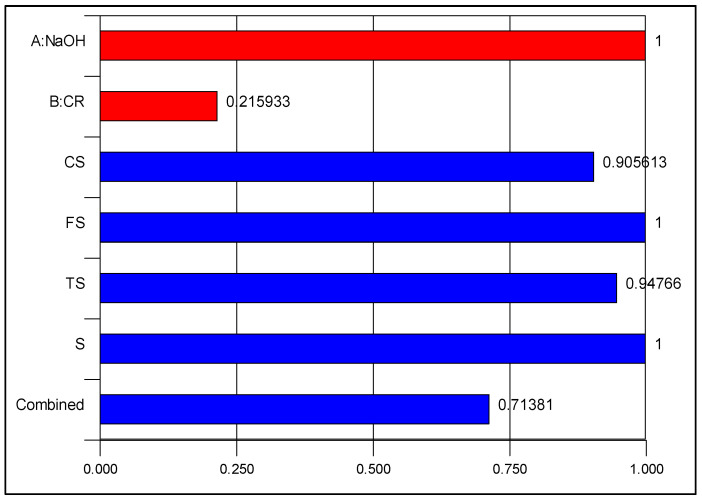
Desirability.

The optimization solution is presented in Figure 16 as ramps showing the optimal values of the input and output factors. The interpretation of the result is that by using 2% CR pretreated by 10% NaOH, a NaOH-CRC having CS, FE, TS, and S of 33.87%, 5.96%, 3.56%, and 4%, respectively as the maximum possible values, can be produced. Figure 17 depicts the desirability values of each variable and the overall desirability value of the multi-objective optimization. The desirability value of 71% indicates a good outcome considering the difficulty of the operation to strike a balance in obtaining optimal values without compromising any of the properties.

### 4.3. Experimental Validation

The optimization was experimentally validated by producing a mix containing the optimized levels of the input factors (CR and NaOH). Three samples were cast for each of the four properties considered as the responses (CS, FS, STS and S) in the RSM analysis. The samples were tested after 28 days and the results are presented together with the predicted results in Table 6. The percentage error between the predicted and the experimental responses was calculated using Equation (5), and the result is also presented in Table 6. As can be observed from the percentage error values, the margin of error for all the responses is within a reasonable range, and hence, this confirms the accuracy of the developed response models.

(5) $$Experimental\;error\;\left(\delta \right)=\left|\frac{Experimental\;value-Predicted\;value}{Predicted\;value}\right|\times 100\%$$

## 5. Conclusions

This research aimed to determine the effect of NaOH pretreatment of CR on the mechanical strengths, ITZ interactions, and drying shrinkage of CRC and develop predictive models and perform optimization using RSM. At the end of the research, the following conclusions can be drawn:

1. It was found that an increase in CR replacement of fine aggregate adversely affects the CS, FS, TS, and S of the NaOH-CRC. However, the rate of strength loss is significantly reduced with NaOH pretreatment. Increases in NaOH pretreatment concentration resulted in a 22%, 44%, and 43% decrease in strength loss for CS, FS, and TS, respectively.
2. The pretreatment significantly reduced the shrinkage. A 63% decrease was reported between a mix containing 10% untreated CR and 10% NaOH-CR. This is due to the enhanced reactivity of the CR with NaOH pretreatment, which results in improved interaction and production of cement hydration products and densification of the ITZ.
3. The FESESM investigations revealed a significantly reduced ITZ with an increase in the NaOH pretreatment. The pretreatment removed the hydrophobic zinc stearate on the CR surface, converting it to sodium stearate, which was washed off during the pretreatment, making the surface more hydrophilic, allowing for better bonding with cement paste and other cement hydration products.
4. Response surface-based models were developed and validated using ANOVA. The models were assessed to be strong for having R^2^ values of 0.9709, 0.7872, 0.9677, and 0.9803 for the CS, FS, TS, and S, respectively. The optimization resulted in an optimal value of 10% and 2% NaOH and CR levels, respectively. At the optimal levels of the input factors, the system predicted values of 33.9 MPa, 6.0 MPa, 3.6 MPa, and 0.04% as the optimal values of CS, FS, TS, and S, respectively, at a desirability index of 71.4%.
5. The performance of rubberized concrete has significantly improved as a result of a better interaction between the CR and the hardened cement paste due to the NaOH pretreatment, and it can now be recommended for structural use in the construction of elements such as foundations.

## Figures and Tables

**Table 1:** Properties and oxide composition of ordinary Portland cement (OPC).

Oxide	CaO	SiO_2_	Fe_2_O_3_	Al_2_O_3_	K_2_O	MgO	SO_3_	P_2_O_5_	TiO_2_	MnO	ZnO	CuO	LOI	Specific Gravity
(%)	82.10	8.59	3.18	2.00	0.72	0.62	2.78	0.46	0.17	0.15	0.03	0.03	2.2	3.15

**Table 2:** Response surface methodology (RSM) developed mixes and materials proportions.

Standard/Mix No.	Run	RSM Input Factors (%)		Materials (Kg/m^3^)				
		A: NaOH	B: CR	NaOH-CR	Fine Agg.	Cement	Coarse Agg.	Water
M1	5	0	0	0.0	850.0	375	1065	178
M5	3	0	5	11.8	812.7	375	1065	178
M3	2	0	10	23.6	775.4	375	1065	178
M7	4	5	5	11.8	812.7	375	1065	178
M9	9	5	5	11.8	812.7	375	1065	178
M8	6	5	10	23.6	775.4	375	1065	178
M6	1	10	5	11.8	812.7	375	1065	178
M2	7	10	5	11.8	812.7	375	1065	178
M4	8	10	10	23.6	775.4	375	1065	178

**Table 3:** Result of analysis of variance (ANOVA).

Response	Source	Sum of Squares	Df	Mean Square	F-Value	*p*-Value > F	Significance
CS (MPa)	Model	237.13	5	47.43	20.01	0.0164	significant
	A-NaOH	39.69	1	39.69	16.75	0.0264	significant
	B-CR	83.81	1	83.81	35.36	0.0095	significant
	AB	0.24	1	0.24	0.10	0.7714	not significant
	A^2^	2.10	1	2.10	0.89	0.4160	not significant
	B^2^	42.75	1	42.75	18.04	0.0239	significant
	Residual	7.11	3	2.37			
	Lack of Fit	1.07	1	1.07	0.35	0.6119	not significant
	Pure Error	6.04	2	3.02			
	Cor Total	244.24	8				
FS (MPa)	Model	1.33	2	0.66	11.10	0.0096	significant
	A-NaOH	0.54	1	0.54	8.97	0.0242	significant
	B-CR	1.06	1	1.06	17.63	0.0057	significant
	Residual	0.36	6	0.060			
	Lack of Fit	0.33	4	0.082	5.64	0.1563	not significant
	Pure Error	0.029	2	0.015			
	Cor Total	1.69	8				
TS (MPa)	Model	1.72	5	0.34	17.95	0.0192	significant
	A-NaOH	0.26	1	0.26	13.32	0.0355	significant
	B-CR	0.88	1	0.88	45.75	0.0066	significant
	AB	0.023	1	0.023	1.21	0.3521	not significant
	A^2^	5.901E−003	1	5.901E−003	0.31	0.6179	not significant
	B^2^	0.50	1	0.50	26.14	0.0145	significant
	Residual	0.058	3	0.019			
	Lack of Fit	0.043	1	0.043	5.97	0.1346	not significant
	Pure Error	0.014	2	7.225E−003			
	Cor Total	1.78	8				
S (mm)	Model	2.07	5	0.41	29.85	0.0092	significant
	A-NaOH	0.70	1	0.70	50.68	0.0057	significant
	B-CR	0.47	1	0.47	34.11	0.0100	significant
	AB	0.011	1	0.011	0.77	0.4459	not significant
	A^2^	0.23	1	0.23	16.68	0.0265	significant
	B^2^	0.11	1	0.11	7.76	0.0687	not significant
	Residual	0.042	3	0.014			
	Lack of Fit	0.030	1	0.030	4.91	0.1571	not significant
	Pure Error	0.012	2	6.025E−003			
	Cor Total	2.11	8				

**Table 4:** Model validation.

Parameters	CS	FS	TS	S
Std. Dev.	1.54	0.24	0.14	0.12
Mean	24.15	5.21	2.54	0.83
C.V. %	6.37	4.70	5.44	14.27
PRESS	-	1.14	-	-
−2 Log Likelihood	23.42	−3.45	−19.93	−22.85
R^2^	0.9709	0.7872	0.9677	0.9803
Adj. R^2^	0.9224	0.7162	0.9138	0.9475
Pred. R^2^	-	0.3231	-	N/A
Adeq. Precision	14.299	8.288	13.504	15.329
BIC	36.60	3.14	−6.74	−9.66
AICc	77.42	7.35	34.07	31.15

Std. Dev.: Standard deviation; C.V.: Coefficient of Variation; PRESS: Predicted residual error sum of squares; R^2^: Coefficient of determination; Adj. R^2^: Adjusted R^2^; Pred. R^2^: Predicted R^2^; Adeq. Precision: Adequate precision; BIC: Bayesian Information Criteria; AICc: Akaike’s Information Criteria.

**Table 5:** Multi-objective optimization criteria and objectives.

Factors		Level		Target	Level of Importance (1–5)
		Lower	Upper		
Input	NaOH (%)	0	10	In range	3
	CR (%)	0	10	Maximize	3
Output	CS (MPa)	17.29	35.60	Maximize	3
	FS (MPa)	4.38	5.91	Maximize	3
	TS (MPa)	2.11	3.64	Maximize	3
	S (%)	0.38	1.91	Minimize	3

**Table 6:** Experimental validation and percentage error.

Response	Predicted	Experimental	δ (%)
CS (MPa)	33.87	36.11	6.6
FS (MPa)	5.96	6.26	5.0
STS (MPa)	3.56	3.32	6.7
S (mm)	0.039	0.041	5.1

## Data Availability

All the data is contained within the article.
